# Does the Endoscopic Surgical Skill Qualification System improve patients’ outcome following laparoscopic surgery for colon cancer? A multicentre, retrospective analysis with propensity score matching

**DOI:** 10.1186/s12957-021-02155-z

**Published:** 2021-02-19

**Authors:** Keisuke Kazama, Masakatsu Numata, Toru Aoyama, Yosuke Atsumi, Hiroshi Tamagawa, Teni Godai, Hiroyuki Saeki, Yusuke Saigusa, Manabu Shiozawa, Norio Yukawa, Munetaka Masuda, Yasushi Rino

**Affiliations:** 1 Department of Surgery, Yokohama City University, 3-9 Fukuura, Kanazawa-ku, Yokohama, 236-0004 Japan; 2 Department of Surgery, Fujisawa Shounandai Hospital, Fujisawa, Japan; 3 Department of Surgery, Yokohama Minami Kyosai Hospital, Yokohama, Japan; 4 Department of Biostatistics, Yokohama City University, Yokohama, Japan; 5 Department of Gastrointestinal Surgery, Kanagawa Cancer Center, Yokohama, Japan

**Keywords:** Endoscopic Surgical Skill Qualification System, Laparoscopic surgery, Colon cancer, Proficiency, Propensity score matching

## Abstract

**Background:**

This study aimed to investigate the short-term and oncological impact of the Endoscopic Surgical Skill Qualification System (ESSQS) by the Japan Society for Endoscopic Surgery on the operator performing laparoscopic surgery for colon cancer.

**Methods:**

This retrospective cohort study was based on medical records from a multicentre database. A total of 417 patients diagnosed with stage II/III colon and rectosigmoid cancer treated with curative resection were divided into two groups according to whether they were operated on by qualified surgeons (*Q* group, *n*=352) or not (NQ group, *n*=65). Through strict propensity score matching, 98 cases (49 in each group) were assessed.

**Results:**

Operative time was significantly longer in the NQ group than in the Q group (199 vs. 168 min, *p*=0.029). The amount of blood loss, post-operative complications, and duration of hospitalisation were similar between both groups. No mortality was observed. One conversion case was seen in the NQ group. The 3-year recurrence-free survival rate was 86.6% in the NQ group and 88.2% in the Q group, which was not statistically significant (log-rank *p*=0.966).

**Conclusion:**

Direct operation by ESSQS-qualified surgeons contributed to a shortened operation time. Under an organised educational environment, almost equivalent safety and oncological outcomes are expected regardless of the surgeon’s qualifications.

## Background

Laparoscopic surgery for colon cancer (LAC) is a popular procedure not only in Japan, but also worldwide, owing to its short-term benefits and acceptable oncological outcomes compared with open surgery [1–4]. In Japan, more than 55% of all surgeries for colon and rectosigmoid malignancy were performed via laparoscopic surgery, according to the National Clinical Database 2018 [5].

The surgical quality of LAC was previously focussed on its clinical impact. It has been reported that high institutional volume decreases intraoperative comorbidities [6] and that surpassing the learning curve improved short-term outcomes [7–13]. In addition, a structured assessment of technical skills performed in LAC was reported to predict complications after surgery [14]. These facts suggest that surgical skill itself is quite important and may theoretically affect the oncological outcomes in LAC.

In 2004, the Japan Society for Endoscopic Surgery (JSES) introduced the Endoscopic Surgical Skill Qualification System (ESSQS), which aimed to improve the technical skills of surgeons, ensure a standardised laparoscopic surgery process, and maintain good outcomes of such procedures performed in Japan. To qualify through this system, a video review of LAC is required. In the review, the preciseness and smoothness of manipulation, and the leadership skills while working with the assistant and laparoscopist are examined, including the effectiveness and oncological feasibility of the standardised procedure, which has low acceptance rates of 20–30% for the colon region.

As for the clinical effect of the ESSQS on qualified surgeons, only a limited number of reports are available, and both short- and long-term outcomes remain unclear. In addition, these reports compare the outcomes of surgeries in which at least one of the participants is a qualified surgeon (SPQ) and that of surgeries in which none of the participants are qualified surgeons (SnPQs). In these reports, the definition of participation of qualified surgeons included not only performing the operation but also participating as an assistant, laparoscopist, or supervisor [15, 16]. Hence, the impact of qualified surgeons participating only as an operator during surgery has never been examined.

Therefore, we undertook this study to clarify the clinical impact of the surgical skill of qualified surgeons who perform the operation during LAC, by comparing between surgeries in which qualified surgeons operated (SOQs) and surgeries in which non-qualified surgeons operated (SnOQs).

## Methods

### Study design

**Fig. 1:**
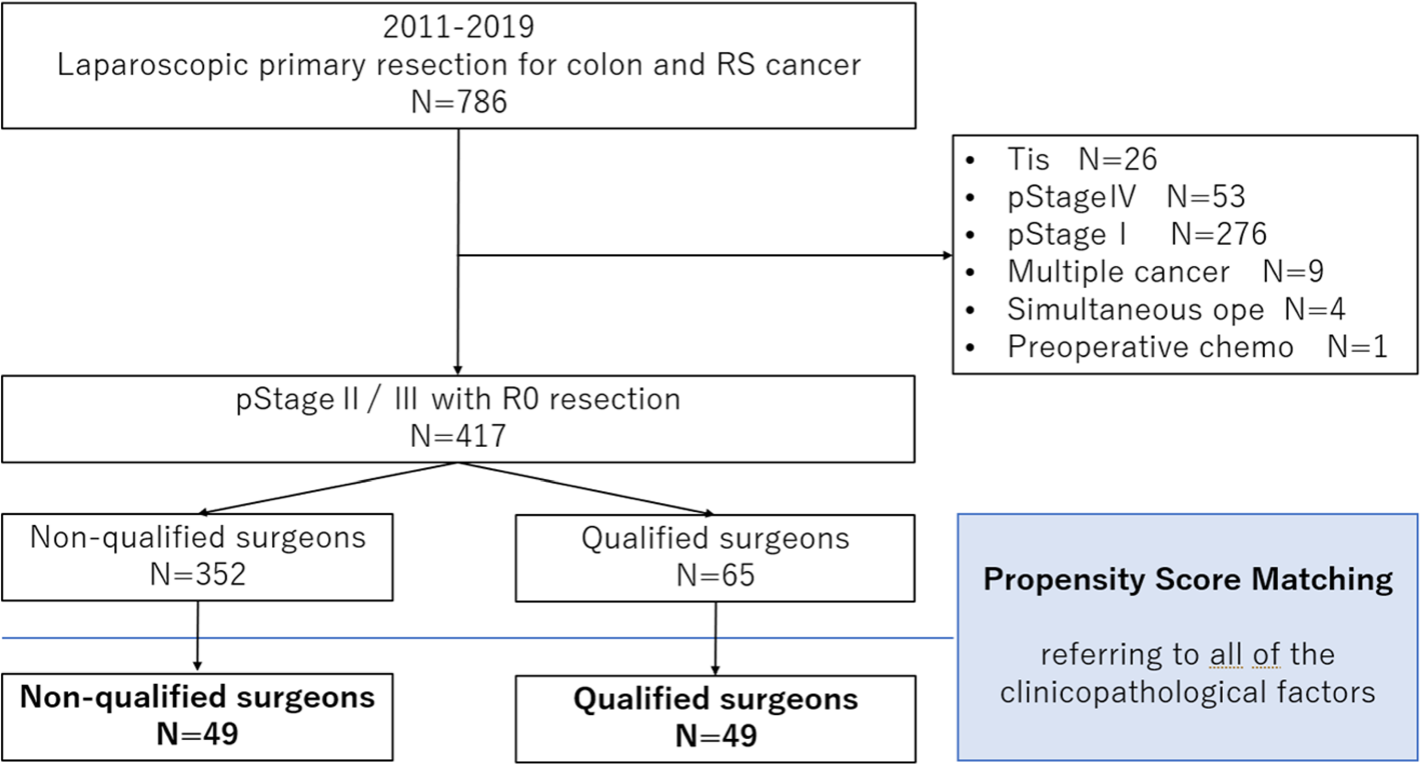
Study design. RS, rectosigmoid; pStage, pathological stage; Simultaneous ope, simultaneous operations on other organs; chemo, chemotherapy; qualified surgeon, surgeon qualified under the Endoscopic Surgical Skill Qualification System

In this retrospective cohort study, the clinical records and database of the Yokohama City University and three group facilities obtained between January 2011 and December 2019 were reviewed. As per these records, a total of 786 colon and rectosigmoid cancer patients underwent LAC. Of these, we excluded 26 patients with tumour in situ (Tis), 53 with pathological stage (pStage) IV, 276 with pStage I, nine with synchronous or multiple cancers, four with simultaneous operations of other organs, and one with preoperative chemotherapy. Finally, 417 patients who had been diagnosed with pStage ll/lll with curative effect were enrolled in this study. Through propensity score matching (PsM), we selected 98 matched patients who were further classified into two groups: patients operated on by non-qualified surgeons (NQ group, *n*=49) and patients operated on by qualified surgeons (Q group, *n*=49) (Fig. 1). In this study, qualified surgeons who only attended as an assistant, laparoscopist, or supervisor during surgery were classified as part of the NQ group; only those qualified surgeons who performed the operation were classified as the Q group.

### Examining the ESSQS

The ESSQS was assessed based on the following criteria: (1) achievements: at least two papers and three presentations on laparoscopic surgery in academic societies; (2) experience: more than 2 years of experience as a general surgeon after certification by the Japan Surgical Society, and at least 20 laparoscopic surgeries demanding advance skills (e.g. colorectal surgery and gastric surgery for cancer) or 50 laparoscopic surgeries demanding basic skills (e.g. cholecystectomy and repairment of inguinal hernia); (3) seminars: attendance at JSES official training seminars such as dry-laboratory on suturing; and (4) video review: review of unedited video and score provided according to the scoring criteria by more than two expert laparoscopic surgeons designated by the JSES. For the colorectal region, sigmoidectomy and high anterior resection of rectum were considered eligible procedures.

### Outcomes of interest

The primary outcome of this study was the 3-year recurrence-free survival (RFS), and the secondary outcomes were short-term outcomes such as operative time, intraoperative blood loss, extent of lymph node dissection, conversion rate, incidence of post-operative complications, and length of post-operative hospitalisation. Operative time is defined as time from skin incision for the first port to completion of all surgical incisions. For the measurement of post-operative surgical complications, the Clavien–Dindo classification was adapted, and the incidence of grade ≥3 complications within 30 days after the operation or during hospitalisation was counted [17].

### Evaluations

All reviewed and evaluated clinicopathological factors from the clinical records and database were as follows: age, sex, American Society of Anesthesiologists performance status (ASA-PS), body mass index (BMI), tumour location, preoperative carcinoembryonic antigen (CEA), carbohydrate antigen 19-9 (CA19-9), preoperative ileus, adjuvant chemotherapy, pStage, tumour diameter, histological type, lymphatic invasion, and vascular invasion. Notation of pathological findings in this study was in accordance with the Japanese Society for Cancer of the Colon and Rectum (JSCCR) guidelines (9th) [18].

### Propensity score matching

One-to-one PsM was applied to all of the patient’s clinicopathological factors, as mentioned above (e.g. pathological stage [II/III], location of tumour [right-sided/left-sided], range of lymphadenectomy [D1/D2 vs. D3], preoperative ileus [yes/no], and adjuvant chemotherapy [yes/no]), to achieve control of the standardised difference under 0.15.

### Operative procedure and follow-up

Ileocecal resection or right hemicolectomy was performed for caecum, ascending colon, and right to middle transverse colon cancer. Left hemicolectomy was selected for left-sided transverse colon and descending colon cancer. For sigmoid and rectosigmoid cancer, sigmoidectomy and high anterior resection were selected. In principle, a five-port setting was utilised. Complete mesocolic excision (CME) was started with the medial approach followed by central vascular ligation (CVL) with lymph node dissection (LND). The appropriate extent of CVL with LND was decided in advance by each surgical team and conference, according to the JSCCR guidelines. A lateral approach was finally added to achieve CME and complete mobilisation of the colon. Functional end-to-end anastomosis at the extra-abdominal field through a nominally extended incision of the umbilical site was selected for reconstruction of the colon. For reconstruction of the rectosigmoid, the double-stapling technique was employed. All of the procedures above were standardised in periodic meetings and were shared with the group facilities.

Medical follow-ups with computed tomography and blood tests were conducted every 6 months for more than 3 years. Colonoscopy was performed 1, 3, and 5 years after surgery.

### Statistical analysis

The clinicopathological parameters were assessed by calculating the median and range, performing the *t* test or Mann-Whitney *U* test for continuous variables, and the proportion and chi-square test or Fisher’s exact test for discrete variables. The Kaplan-Meier method was used to estimate the RFS. Survival was compared between the two groups using the log-rank test. For statistical analyses, the authors used EZR, a graphical user interface for R version 2.13.0 (The R Foundation for Statistical Computing, Vienna, Austria), and the R software version 3.5.1. More precisely, EZR is a modified version of R commander designed to add statistical functions frequently used in biostatistics. Two-sided *p* values were calculated, and *p* values under 0.05 were considered to be statistically significant.

## Results

### Clinicopathological background of the enrolled patients before and after PsM

The clinicopathological background of the enrolled patients before and after PsM with standardised difference score are summarised in Table 1. Before PsM, 352 patients were classified into the NQ group and 65 into the Q group, respectively. As for clinical features, the median age (70 vs. 73 years) and BMI (22.5 vs. 23.4 kg/m^2^) were higher in the Q group. Moreover, the proportion of patients with Class 3 ASA-PS was higher in the Q group than in the NQ group (13.6 vs. 24.6%) and the preoperative CA19-9 level tended to be higher in the Q group (7.0 vs. 11.9 ng/ml). Other clinical features such as sex, tumour location, preoperative ileus, previous laparotomy, adjuvant chemotherapy, and preoperative CEA were almost similar in both groups. Regarding pathological features, the proportion of patients with histologically undifferentiated cancer tended to be higher in the Q group (5.4 vs. 12.3%). No difference was observed for pStage, tumour diameter, lymphatic invasion, or vascular invasion.

**Table 1:** Patient characteristics

	Before PsM			After PsM			
	NQ group	Q group	*p* value	NQ group	Q group	*p* value	std
	(*N*=352)	(*N*=65)		(*N*=49)	(*N*=49)		
Age, years	70 (24–98)	73 (46–89)	0.008	72 (38–90)	73 (46–89)	0.929	0.070
Sex			0.422			0.686	
Male	181 (51.4%)	37 (56.9%)		24 (49.0%)	27 (55.1%)		− 0.123
Female	171 (48.6%)	28 (43.1%)		25 (51.0%)	22 (44.9%)		0.123
BMI	22.5 (14.2-38.5)	23.4 (15.4-38.1)	0.035	22.7 (14.4–30.2)	23.3 (15.4–37.7)	0.481	− 0.143
ASA-PS			0.037			0.245	
Class 1, 2	304 (86.4%)	49 (75.4%)		39 (79.6%)	38 (77.6%)		0.050
Class 3	48 (13.6%)	16 (24.6%)		10 (20.4%)	11 (22.4%)		− 0.050
Tumour location			0.495			0.834	
Right-sided	147 (41.8%)	24 (36.9%)		19 (38.8%)	17 (34.7%)		0.085
Left-sided	205 (58.2%)	41 (63.1%)		30 (61.2%)	32 (65.3%)		− 0.085
Preoperative ileus			0.349			1	
Yes	57 (16.2%)	7 (10.8%)		6 (12.2%)	7 (14.3%)		− 0.060
No	295 (83.8%)	58 (89.2%)		43 (87.8%)	42 (85.7%)		0.060
Previous laparotomy			0.259			1	
Yes	75 (21.3%)	18 (27.7%)		12 (24.5%)	12 (24.5%)		0
No	277 (78.7%)	47 (72.3%)		37 (75.5%)	37 (75.5%)		0
Adj chemo			0.891			1	
Yes	146 (41.5%)	26 (40.0%)		20 (40.8%)	21 (42.9%)		− 0.041
No	206 (58.5%)	39 (60.0%)		29 (59.2%)	28 (57.1%)		0.041
CEA, ng/ml	3.2 (0.3–79.1)	3.0 (0.6–44.0)	0.499	2.7 (0.5–43.9)	3.1 (0.6–44.0)	0.529	− 0.015
CA19-9, ng/ml	7.0 (1.0–644.5)	11.9 (1.0–1933.0)	0.056	8.2 (1.0–644.5)	11.9 (1.00–184.0)	0.189	− 0.137
pStage			1			0.840	
pStage II	163 (46.3%)	30 (46.2%)		23 (46.9%)	25 (51.0%)		− 0.082
pStage III	189 (53.7%)	35 (53.8%)		26 (53.1%)	24 (49.0%)		0.082
Histological type			0.052			0.715	
Differentiated	333 (94.6%)	57 (87.7%)		46 (93.9%)	44 (89.8%)		0.149
Undifferentiated	19 (5.4%)	8 (12.3%)		3 (6.1%)	5 (10.2%)		− 0.149
Tumour diameter, mm	40.0 (2.0–117.0)	45.0 (10.0–110.0)	0.131	40.0 (12.0–117.0)	45.0 (10.0–90.0)	0.270	− 0.144
Lymphatic invasion			0.282			0.840	
Positive	179 (50.9%)	38 (58.5%)		24 (49.0%)	26 (53.1%)		− 0.082
Negative	173 (49.1%)	27 (41.5%)		25 (51.0%)	23 (46.9%)		0.082
Vascular invasion			0.132			0.684	
Positive	211 (59.9%)	32 (49.2%)		29 (59.2%)	26 (53.1%)		0.124
Negative	141 (40.1%)	33 (50.8%)		20 (40.8%)	23 (46.9%)		− 0.124

Continuous variables are presented as medians with ranges; discrete variables are presented as numbers with percentages

*NQ group* non-qualified surgeon group, *Q group* qualified surgeon group, *PsM* propensity score matching, *BMI* body mass index, *ASA-PS* American Society of Anesthesiologists performance status, *Adj chemo* adjuvant chemotherapy, *std* standardised difference

After PsM, clinicopathological factors were well balanced between the groups.

### Short-term outcomes of the matched population

Table 2 displays the short-term outcomes. Regarding intraoperative outcomes, operative time was significantly longer in the NQ group than in the Q group (199 vs. 168 min, *p*=0.029). The amount of blood loss was almost similar (10 vs. 10 ml, *p*=0.961). D3 LND was performed in 75.5% of the NQ group and 83.7% of the Q group (*p*=0.453). Only one case (2.0%) in the NQ group required conversion to open surgery. This was done because of the perforation of the small bowel caused by forced lifting that was carried out to get good visualisation which was hindered by the visceral fat in a higher-BMI patient (26.8 kg/m^2^), whereas no conversion was needed in the Q group.

**Table 2:** Short-term outcomes

	NQ (*N*=49)	*Q* (*N*=49)	*p* value
Operative time, min	199 (112–287)	168 (95–304)	0.029
Blood loss, ml	10 (1–800)	10 (1–560)	0.961
Extent of LND, D1/D2 vs. D3			0.453
D1/D2	12 (24.5%)	8 (16.3%)	
D3	37 (75.5%)	41 (83.7%)	
Conversion to open surgery	1 (2.0%)	0	1
Complication, grade ≥3	3 (6.1%)	3 (6.1%)	1
Anastomotic leakage	2 (4.1%)	0	
Pneumonia	1 (2.0%)	0	
Ileus	0	1 (2.0%)	
Port site hernia	0	1 (2.0%)	
Wound infection	0	1 (2.0%)	
Mortality	0	0	
Hospitalisation, days	9 (6–74)	10 (5–37)	0.927

Continuous variables are presented as medians with ranges; discrete variables are presented as numbers with percentages

*NQ group* non-qualified surgeon group, *Q group* qualified surgeon group, *LND* lymph node dissection, *CD* Clavien–Dindo classification, *SSI* surgical site infection

Regarding post-operative outcomes, grade of complications ≥3 according to the Clavien–Dindo classification was observed in three cases (6.1%) in each group. In the NQ group, two cases were that of anastomotic leakage (in right hemicolectomy and sigmoidectomy) and one case was that of pneumonia. In the Q group, one case each of ileus, port site hernia, and surgical site infection were observed. No mortality was observed in either group. The duration of hospitalisation was similar between the two groups (9 vs. 10 days, *p*=0.927).

### RFS and details of recurrence sites

**Fig. 2:**
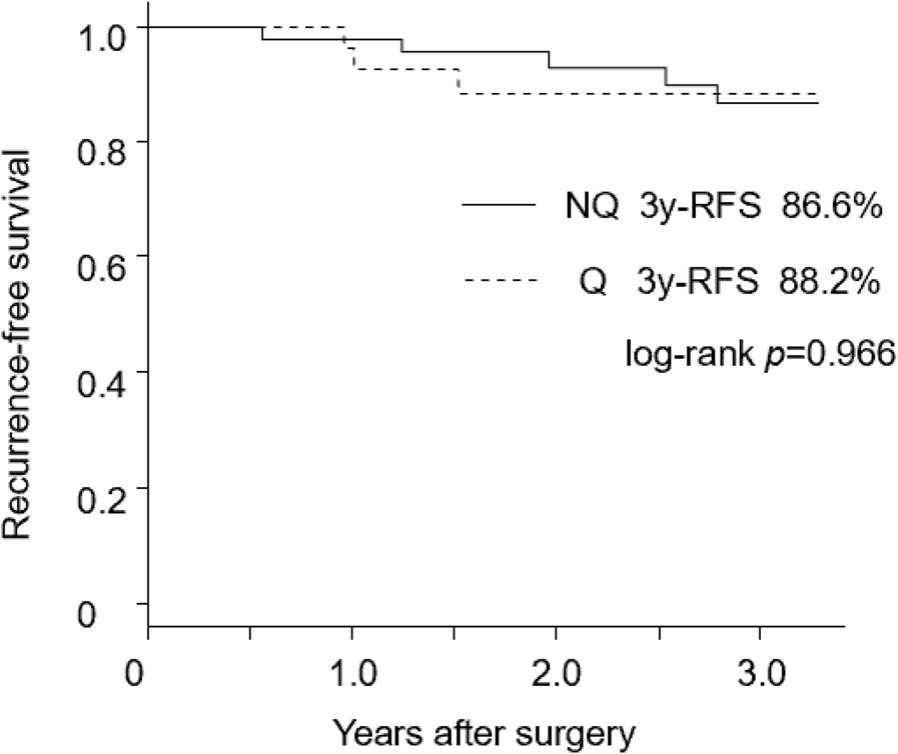
Recurrence-free survivals in the NQ and Q groups. NQ group, non-qualified surgeon group; Q group, qualified surgeon group; 3y-RFS, recurrence-free survival rate at 3 years after operation

The median follow-up duration was 24.5 months in the entire cohort. The Kaplan-Meier curves for the 3-year RFS are presented in Fig. 2 (log-rank *p*=0.966). The 3-year RFS rate was almost similar between both groups: 86.6% (95% confidence interval [CI]: 70.4–94.5%) in the NQ group and 88.2% (95% CI: 67.5-96.1%) in the Q group.

Details of the recurrence sites are shown in Table 3. Recurrence was observed in five cases (10.2%) in the NQ group and three cases (6.1%) in the Q group (*p*=0.715), of which liver metastasis was most frequently observed. Both groups had no local recurrence.

**Table 3:** Details of recurrence

	NQ (*N*=49)	Q (*N*=49)	*p* value
Recurrence (%)	5 (10.2%)	3 (6.1%)	0.715
Liver	3 (6.1%)	2 (4.1%)	
Peritoneum	2 (4.1%)	0	
Lung	0	1 (2.0%)	

Discrete variables are presented as numbers with percentages

*NQ group* non-qualified surgeon group, *Q group* qualified surgeon group

## Discussion

The ESSQS is a unique qualification system in terms of evaluating the surgical technique itself, with a low acceptance rate. Recently, participation of ESSQS-qualified surgeons in LAC was reported to be beneficial [15, 16]. However, to our knowledge, the clinical impact of qualified surgeons who actually perform the operation during surgery has not been addressed in previous studies. In the current study, the patient cohort was classified into two groups based on whether the operation was performed directly by a qualified surgeon or not, and the short- and long-term outcomes of SOQs and SnOQs were compared through PsM. As a result, SOQs had a statistically shorter operative time than SnPQs, whereas blood loss, rate of post-operative complications, and conversion rate were similar between the two groups, and ESSQS did not affect the long-term outcomes.

Globally, the most widely accepted framework of surgical innovation is the IDEAL paradigm advocated by McCulloch, where the roles of a surgeon are divided into 4 phases; phase1 ‘Innovator’, phase2 ‘Pioneer’, phase3 ‘Early adopter’. and phase4 ‘Established practice’ [19, 20]. According to the concept of ESSQS, qualified surgeons seem to be categorized in phase3, and from the view of a report by Gumbs et al., non-qualified surgeons in phase4 as ‘newly trained surgeons’ [21]. And some previous studies focussing on the relationship between proficiency level and post-operative outcome mainly divided surgeons into the two groups by the term ‘trainers and trainees’, which also suggest ‘surgeon of phase3 and phase4’, respectively.

In the reports of the early 2000s, it was generally considered that proficiency level affected short-term outcomes such as bleeding, conversion rate, and post-operative complications. For example, Daetwiler et al. reported that cases of LAC performed by trainees had a greater amount of bleeding and a higher conversion rate than in those performed by trainers [22]. Moreover, Philipp et al. demonstrated that in their retrospective cohort study of 1316 patients who underwent LAC, a multivariate analysis selected operation by a trainee to be an independent risk factor for post-operative complications [23]. However, more recently, many reports in the 2010s support the idea that the safety of LAC performed by a trainee is equivalent to that when the procedure is performed by a trainer [8, 9, 24–26]. For example, Maeda et al. showed that operation by a trainee was not selected as an independent risk factor for overall morbidity in their retrospective cohort study of 204 patients who underwent LAC [8]. Additionally, according to a systematic review by Kelly et al., no difference was observed in terms of conversion, surgical complications, and mortality in surgeries performed by trainers and trainees [26].

Why could the trainee have comparable outcomes to the trainer? The possible explanation is as follows. First, perhaps as the most important reason, the importance of standardised procedures has become widely recognised [9, 27–30], and trainees have been able to practice such procedures through organised educational systems. This leads to a shortened learning curve and preserves the operative quality homogeneously [10]. Second, manipulation and situational training became very effective through virtual simulation systems and educational programmes on multimedia, which were not widely available in the early 2000s [31–33], leading to improved personal skills and knowledge. Third, the participation of experienced surgeons as supervisors became increasingly popular in clinical practice [8, 16]. The group facilities of our department maintain organised educational systems such as regularly scheduled conferences for video review to provide feedback from a senior surgeon and share the standardised procedure. The results of the present study are consistent with the previous concept that the short-term outcome was preserved regardless of the surgeon’s proficiency level in organised educational facilities. Furthermore, as for the safety, El Amrani et al. reported that a facility volume was associated with mortality of gastrointestinal surgery, suggesting that not only operative technique but also the quality of perioperative care and availability of equipment of a facility such as intensive-care units and interventional radiography are important. These may mask the differences in mortality and complication rates among surgeons [34].

With regard to operative time, although a previous report showed that surgeon experience and operation time are irrelevant [35], most investigators suggest the superiority of the trainer-to-trainee approach over other factors [8, 9, 24, 36]. Experienced surgeons generally have more opportunities to operate in difficult cases, which require longer operation time, than do novice surgeons. Therefore, matching of patients’ backgrounds by PsM was adapted in this study, and as a result, the advantage of ESSQS was clearly demonstrated in terms of operative time.

In this study, the 3-year RFS was not statistically different between SOQs and SnOQs. As for the impact of the surgeon’s skill on long-term outcomes in LAC, few studies have been conducted previously. Henry et al. retrospectively compared the 2-year recurrence rates of those who underwent LAC performed by a trainer (*n*=125) and by a trainee (*n*=56) at a single centre. Consequently, the local recurrence rate was 0% in the trainee group and 0.5% in the trainer group *(p*=1.000), and metastatic recurrence was observed in 0% vs. 3.0% (*p*=0.553), and there were no statistically significant differences [9]. This may theoretically come from the same reason for short-term outcomes—that is, LAC by trainees can promise oncological safety in organised educational teams, and our results are consistent with their report.

Some limitations should be noted when interpreting our results. First, this is a retrospective cohort study with a limited sample size. The statistical power might be insufficient due to the small sample size. Second, through PsM, many older patients and those with a higher BMI in the NQ group were excluded from this study. This suggests that the patients’ backgrounds in the current study differ from those seen in clinical settings. Third, in current study, the details of laparoscopists and additional assistants were not examined. Participation of qualified surgeons as laparoscopists or supervisors could mask the differences of outcomes of the two groups. A fourth limitation is regarding the definitions of the trainee and trainer. In this section, we used the terms trainer and trainee instead of Q and NQ, but these definitions are not fixed and differ between past reports.

## Conclusion

In conclusion, direct operation by ESSQS-qualified surgeons contributed to a shortened operation time. Under an organised educational environment, almost equivalent safety and oncological outcomes are expected regardless of the surgeon’s qualifications.

## Data Availability

The datasets generated and analysed during the current study are not publicly available because data transfer to third party has not been referred to in the protocol.

## Abbreviations

ESSQS: Endoscopic Surgical Skill Qualification System
LAC: Laparoscopic surgery for colon cancer
JSES: Japan Society for Endoscopic Surgery
SPQ: Surgery in which at least one of the participants is a qualified surgeon
SnPQ: Surgery in which none of the participants are qualified surgeons
SOQ: Surgery performed by qualified operator surgeon
SnOQ: Surgery performed by non-qualified operator surgeon
PsM: Propensity score matching (PsM)
NQ: Group of patients operated on by non-qualified surgeons
Q: Group of patients operated on by qualified surgeons
RFS: Recurrence-free survival
ASA-PS: American Society of Anesthesiologists performance status (ASA-PS)
BMI: Body mass index
CEA: Carcinoembryonic antigen (CEA)
CA19-9: Carbohydrate antigen 19-9
JSCCR: Japanese Society for Cancer of the Colon and Rectum
CME: Complete mesocolic excision
CVL: Central vascular ligation
LND: Lymph node dissection
